# Simulation and On-Site Detection of the Failure Characteristics of Overlying Strata under the Mining Disturbance of Coal Seams with Thin Bedrock and Thick Alluvium

**DOI:** 10.3390/s24061748

**Published:** 2024-03-08

**Authors:** Qunlei Zhang, Jianping Guo, Xiaowei Lu, Kunpeng Ding, Ruifu Yuan, Decai Wang

**Affiliations:** 1 School of Civil Engineering and Transportation, North China University of Water Resources and Electric Power, Zhengzhou 450045, China; zhangqunlei@ncwu.edu.cn (Q.Z.); wangdecai@ncwu.edu.cn (D.W.); 2 School of Energy Science and Engineering, Henan Polytechnic University, Jiaozuo 454003, China; gjp@home.hpu.edu.cn (J.G.); hnlgdkp@163.com (K.D.); 3 School of Safety Engineering, China University of Mining and Technology, Xuzhou 221116, China; luxiaowei@hpu.edu.cn

**Keywords:** thin bedrock, thick alluvium, clay aquiclude, on-site detection, simulation

## Abstract

When mining deep coal seams with thin bedrock and thick alluvium, the collapse and fracture of thin bedrock layers may cause geological disasters, such as water inrush and sand inrush of the mining face. Comprehensively obtaining the response data of coal mining and reasonably analyzing the failure characteristics of overlying strata are helpful in guiding safe production. In this study, the caving zone heights of overlying strata are obtained by field detection during layered mining. Then, the caving zone heights during the once-full-height mining are evaluated by theoretical analysis. Further, the force and failure characteristics of coal–rock structures under different mining conditions are compared by the simulation detection and analysis. Finally, the results of on-site observation, theoretical analysis, and simulation detection are compared and discussed, and an optimized mining technology is proposed to ensure safe mining. The research shows the caving zone heights of on-site and simulation detections are, respectively, 14.65 m and 13.5 m during bottom-layer mining, which is larger than the caving zone heights of the top-layer coal mining. During once-full-height mining, the maximum caving zone height of simulation detection is 21 m, which is in between two standard results. For the mechanical responses of an aquiclude clay layer under thick loose alluvium, the maximum disturbance displacement of clay aquiclude is 5.8 m during layered mining, which is slightly larger than the disturbance displacement of once full-height mining; however, the maximum stress of the clay layer is 25 MPa during once-full-height mining, which is larger than the maximum stress of clay layer during layered mining. For the clay aquiclude failure, the clay layer during layered mining is in the deflection deformation area, and there is no obvious fracture structure to inrush the water and sand of thick loose alluvium; however, the clay layer during once-full-height mining is prone to produce obvious fracture structure. Therefore, the layered mining technology can effectively reduce and prevent the water/sand inrush disaster of mining working face.

## 1. Introduction

A considerable part of proven coal reserves in the eastern and central mining areas of China are under thick and loose geological conditions. During mining coal seams with thick alluvium and thin bedrock, the failure development of overlying strata affects the mechanical behavior of rock mass, thereby affecting the stability of overburden structures, and the separated layers and fractures of overlying strata form the main water-inflow channels of coal mining face. Mining practices show that thinner bedrock failure easily generates geological disasters such as water inrush and sand burst, which pose a threat to the safety production of coal mines [1]. Therefore, it is necessary to analyze the failure evolution characteristics of thin bedrock and thick alluvium during coal mining.

Roof strata failure is the main cause of water inrush and roof collapse accidents in the mining face. Scholars have performed a lot of research on overlying strata caving and fracture evolution during coal mining. Jira’nkova [2] studied the phenomenon of surface subsidence during coal mining, proposed a method for evaluating the failure of rigid strata overlying coal seams, and believed the characteristics of surface subsidence during mining can be used to identify the failure of rigid overlying strata and to recognize risks in coal mining engineering. Paul et al. [3] studied the stress caused by the coal mining process and the internal stress distribution mode, which makes roof collapse more likely to occur at the intersection of coal mine roadways, especially in the fine column mining method. Liu et al. [4] divided the overburden rock failure into a caving zone, fracture zone, and bending subsidence zone in the vertical direction. Song et al. [5] combined the elastic thin plate and key layer theories to study the distribution characteristics of rock fractures under mining influence, and believed that both side failures of the mining field were larger than the middle failure of the mining field. Through the rock beam structure theory, Xu et al. [6] studied the development of mining-induced fissures in overlying strata under water, and believed that the fissure density near the open-off cut side was greater than that near the stopping line side. Zingano and Andrade [7] studied roof failure and failure mechanism of coal mining intersection through field investigation, geological exploration, and numerical simulation analysis. Yi et al. [8] found that the height of the water-conducting fractured zone will develop into a loose layer after mining a thick coal seam with thin bedrock, and the overlying strata may only exist in two zones (caving zone and fractured zone) or one belt (caving zone). Sun et al. [9] found that the number and adjacent spacing of overlying strata would be greater when the overlying strata thickness was larger. Wang et al. [10] considered that a subsidence basin would be formed on the interface between the bedrock and loose layer as the rock arch structure was destroyed, and the upward propagation of subsidence space caused the surface subsidence.

With the development of sensing technology, the deformation and failure characteristics of overlying strata are investigated by on-site detection. Mishra et al. [11] developed a measurement sensor using vibrating wire (VW), which can realize real-time monitoring of geological conditions. The sensor is consistent with the measurement results of traditional measurement units, which can avoid damage and underground coal mine accidents, and has wide application potential. Bai et al. [12] determined mine damage and induced cracking through drilling tests, and established an empirical relationship between the failure zones in the vertical and horizontal directions. Zheng et al. [13] used the deep borehole stress meters to evaluate the fracturing effect of the hard roof after the ultra-deep borehole hydro-fracturing. Zhu et al. [14] used passive velocity tomography and acoustic emission to study the internal microfractures and energy evolution of coal samples during true-triaxial compression. Zhang et al. [15] used stress sensors to analyze the stress changes of the goaf, the soft mold-filling body, and single pillars. Yang et al. [16] performed physical modeling experiments to analyze the structural characteristics and stress field variation of the overlying strata during multiple coal seam mining. Hu et al. [17] used the distributed optical fiber sensor technology to monitor the roof strata movement, to grasp the movement law of roof strata and make it serve for production. Mekhtiev et al. [18] introduced the new method and new equipment of ground control, and put forward a simple design scheme of optical fiber displacement sensor, which improved the ground control and the safety of mining. By comparing the aperture of the spot, the geotechnical conditions of the roadway are identified. Du et al. [19] used fiber Bragg grating (FBG) and distributed optical fiber (BOTDA) methods to monitor the stability of mining overburden. With the development of computer technology, simulation has also developed into an important research method for coal seam mining with thick loose layers and thin bedrock [20]. Fang et al. [21] used a UDEC simulation to study the law of overlying rock movement, and found that the combination of thick clay layer and thin bedrock can form a stable structure. For the mining of thick coal seams with ultra-thin bedrock and thick loose layers, Du et al. [22] studied the breaking mechanism and the fracture development of the overlying rock, and found that there was no basic roof and key rock layer above the coal seam, and the stress arch was formed in the advancing process of working face. Yang et al. [23] established a numerical model to analyze the deformation and failure law of overlying strata during the mining process, and proposed a calculation formula of the height of the water-conducting fractured zone. Yu et al. [24] studied the law of surface movement and deformation under the mining condition of a deep coal seam with thick loose layers and thin bedrock. The above references systematically investigated the overlying rock failure and surface subsidence under the once full-height mining technology. However, there are few studies on the influence of different mining technologies on the aquiclude clay layer above the thin bedrock.

Generally, the separation and collapse characteristics of overlying strata can be observed by the on-site sensor detection. However, due to the complexity of underground mining, it is difficult to obtain comprehensive data by only using sensor and imaging equipment. Currently, combining on-site detection, numerical simulation, and theoretical analysis has become an effective means by which one can reasonably study the separation and collapse characteristics of overlying strata; moreover, the mechanical failure and water conductivity properties of aquiclude clay layer can be also effectively analyzed. Corresponding research results can guide and ensure mining safety.

This paper takes the deep coal seam with thin bedrock and thick alluvium as the research background, the separation and collapse of overlying strata under the mining disturbance are investigated, and the water/sand inrush disaster of the mining face is evaluated. Concretely, the theoretical formulas and field detection are carried out to obtain the collapse and fracture zone height under different mining technologies. Simulation detection and analysis are used to investigate the force and failure characteristics of coal–rock layers under different conditions (mining technologies and working face lengths). Further, the mechanical responses of the clay layer above bedrock layers are emphatically analyzed to evaluate the water-resisting and sand-resisting abilities. Finally, for different study methods (on-site detection, theoretical analysis, and simulation), the response data of coal mining under different mining technologies are compared and discussed, and an optimized mining technology is proposed to ensure safe production.

## 2. On-Site Detection and Theoretical Analysis of Caving Zone Height

### 2.1. Engineering Background

**Figure 1:**
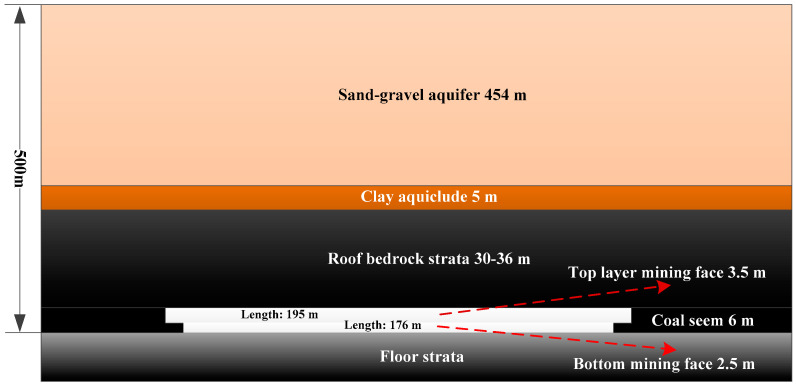
Schematic diagram of engineering background.

For a mine, the buried depth of a mining coal seam is about 500 m, and the average thickness of the coal seam is 6 m. The specific engineering conditions of the research background are shown in Figure 1.

Above the coal seam, the bedrock thickness is 30–36 m, and the thickness of the aquiclude clay layer above the bedrock is about 5 m. The thick sand layer and gravel aquifer are above the clay layer. The layered mining method is adopted in the working face of coal mine. As the upper layered coal seam is mined, the mining height is 3.5 m, the inclined length of the working face is 195 m, and the mining advancing length is 437–476 m. The ground elevation of the mining face is +81~+83 m, and the corresponding underground elevation is −386~−454 m. The comprehensive mechanized longwall mining method is adopted, and the caving method is used to deal with the goaf. As the bottom layered coal is mined, and the mining height is 2.5 m, the inclined length of the working face is 176 m, and the mining advancing length is 454–490 m.

### 2.2. The Field Detection of Caving Zone Height during the Top-Layer Coal Mining

For the top-layered coal mining, the 11,071 working face was the end of mining in March 2012. The caving zone height of the roof strata was detected by ground drilling observation. The detected process and results are as follows: the arrangement scheme of drilling structure is as follows: 0~11.99 m, aperture 311 mm, φ219 mm casing; 11.99~358.71 m, aperture 215 mm, 358.71~500.20 m, aperture 190 mm, φ159 mm waterproof casing is 500.20 m; the aperture of 500.20~547.35 m is 133 mm, which is a bare hole. Actual completion results of drilling detection are shown in Table 1.

According to the ground drilling observation, the formula to calculate the caving zone height [25] is:

(1) $$H_{k}=H-M-H_{1}+W,$$

where *H_k_* is the collapse zone height, m; *H* is the vertical depth from the coal seam floor to the orifice, m; *M* is the coal seam thickness at the drilling position, m; *H*_1_ is the vertical depth from the caving zone top to the orifice, m; and *W* is the compression values of fractured zone strata during drilling observation, m.

In the field measurement, the vertical depth from the coal seam floor to the orifice is 566.5 m, the coal seam thickness at the drilling position is 6.4 m, the vertical depth from the caving zone top to the orifice is 547 m, the compression values of fractured zone strata are taken as 0 m, according to the above formula, and the caving zone height is 13.1 m.

### 2.3. The Field Detection of Caving Zone Height during the Bottom-Layer Coal Mining

**Figure 2:**
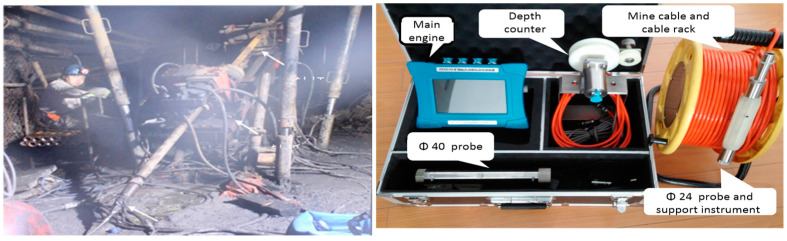
Field drilling and borehole observation.

For bottom-layered coal mining, the 11,072 working face is located below the 11,071 working face, and two roadways of the 11,072 working face are arranged inside 10 m of upper mining face roadways. The stopping position is the same, and the mining end is in April 2021. To detect the caving zone height of roof strata after the bottom coal mining, underground drilling detection is conducted, and the upward inclined boreholes are drilled in the roof strata above the goaf, the ZKXG100 mine drilling imaging device is used to observe the boreholes, as shown in Figure 2.

In this field detection, four boreholes were used to measure the roof caving zone height. The observation results are shown in Table 2. From Table 2, after the bottom-layer coal mining, the caving zone heights of roof strata are an average of 14.65 m.

### 2.4. Theoretical Value of Caving Zone Height during Once Full-Height Coal Mining

In China, the empirical formulas of caving zone height suitable for thick coal seam mining have been summarized based on a large of field observations. In the standard for coal pillar retention and coal mining in buildings, water, railways, and main roadways [26], the empirical formula of caving zone height for the medium hard roof strata is

(2) $$H_{k}=\frac{100\sum M}{4.7\sum M+19}\pm 2.2,$$

For the soft-weak roof strata, the empirical formula of caving zone height after coal mining is

(3) $$H_{k}=\frac{100\sum M}{6.2\sum M+32}\pm 1.5,$$

The roof strata of this study are between medium-hard and soft-weak strata, and the coal thickness is 6 m. According to Equations (2) and (3), the caving zone height is 7.2–14.9 m.

In the evaluation standard for hydrogeological, engineering geological, and environmental geological exploration of coal beds, the empirical formulas of caving zone height suitable for thick coal seam mining are also put forward [27].

For the medium hard roof strata, the empirical formula of caving zone height after coal mining is

(4) $$H_{k}=\left(3\sim 4\right)\sum M,$$

For the soft-weak roof strata, the caving zone height after coal mining is

(5) $$H_{k}=\left(1\sim 2\right)\sum M,$$

According to the above calculation of Equations (4) and (5), as the thickness of the coal seam is 6 m, the caving zone height is 6–24 m.

## 3. Simulation Detection and Analysis of the Overlying Strata Failure

### 3.1. Simulation Method

**Figure 3:**
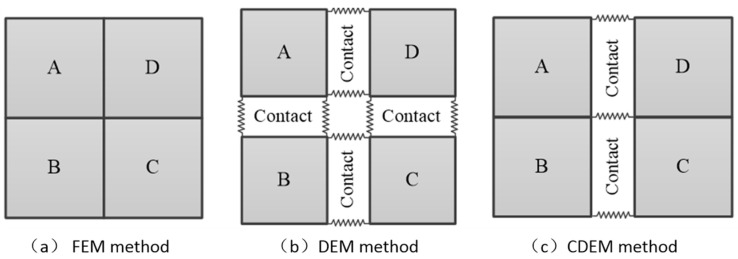
Diagrams for different numerical methods [28].

In this paper, the continuous–discontinuous element method (CDEM) is used to study the failure process of coal–rock strata [28]. CDEM couples with the finite element method and discrete element method, and the numerical model is composed of block element and contact element, as shown in Figure 3. The block model is composed of finite elements, which can characterize the continuous mechanical responses such as elasticity, plasticity, and damage. The contact elements are used in the block interfaces, which can characterize the discontinuous characteristics such as fracture, slip, and collision of materials and structures. Based on this, GDEM commercial software (1.0.30.1730) was developed by the Institute of Mechanics, Chinese Academy of Sciences, which has been successfully applied in mining and other projects.

Based on the dynamic explicit incremental algorithm, the control equations of CDEM are shown below:

(6) $$\begin{array}{c} {}[M]\{\ddot{u}\}+[C]\{\dot{u}\}+[K]\{u\}=\{F{\}}_{ext}\\ \{F{\}}_{ext}=\{F{\}}_{s}+\{F{\}}_{t}, \end{array}$$

where $\left[M\right]$ is the node mass matrix; $\left[C\right]$ is the node damping matrix; $\left[K\right]$ is the node stiffness matrix; $\left\{u\right\}$ is the node displacement vector; ${\left\{F\right\}}_{ext}$ is the vector of external forces vector; ${\left\{F\right\}}_{s}$ is the contact force; and ${\left\{F\right\}}_{t}$ is the external loading force.

The incremental method is used to calculate the stress and deformation of the block element in the continuous calculation area:

(7) $$\left.\begin{array}{c} {\left\{\Delta \xi \right\}}_{i}={\left[B\right]}_{i}\cdot {\left\{\Delta u\right\}}_{e}\\ {\left\{\Delta \sigma \right\}}_{i}={\left[D\right]}_{i}\cdot {\left\{\Delta \xi \right\}}_{i}\\ {\left\{\Delta \sigma^{n}\right\}}_{i}={\left\{\sigma^{0}\right\}}_{i}+{\left\{\Delta \sigma \right\}}_{i}\\ {\left\{F_{d}\right\}}_{e}=\sum_{i=1}^{N}{{\left[B\right]}_{i}}^{T}\cdot {\left\{\sigma^{n}\right\}}_{i}\cdot \omega_{i}\cdot J_{i} \end{array}\right\},$$

where ${\left[B\right]}_{i}$, ${\left\{\Delta \xi \right\}}_{i}$,${\left\{\Delta \sigma \right\}}_{i}$,${\;\omega }_{i}$, and $J_{i}$ are the node strain matrix, node incremental strain vector, node incremental stress vector, integral coefficient, and Jacobian determinant value of calculation element; ${\left\{\Delta \sigma^{n}\right\}}_{i}$ and ${\left\{\sigma^{0}\right\}}_{i}$ is the total stresses at the current and previous calculation steps; ${\left[D\right]}_{i}$,$\;{\left\{\Delta u\right\}}_{e}$,$\;{\left\{F_{d}\right\}}_{e}$ are element elastic matrix, node incremental displacement vector and node force vector; and *N* is number of Gaussian points.

An incremental method is also used to calculate the normal and tangential contact force of the interface element in the discontinuous calculation area. The calculation formulas are:

(8) $$\left.\begin{array}{c} F_{n}(t_{1})-F_{n}(t_{0})=K_{n}\cdot A_{c}\cdot \Delta u_{n}\\ F_{s}(t_{1})-F_{s}(t_{0})=K_{s}\cdot A_{c}\cdot \Delta u_{s} \end{array}\right\},$$

where *F_n_* and *F_s_* are the normal force and tangential force of contact elements, respectively; *k_n_* and *k_s_* are the normal stiffness and tangential stiffness, respectively; *A_c_* is the contact element area; Δ*u_n_* and Δ*u_s_* are the normal relative displacement and the tangential relative displacement, respectively.

In the simulation, the tensile failure criterion is used to calculate and corrected the normal stress of contact element:

(9) $$\begin{array}{c} If\;F_{n}(t_{1})-\sigma (t_{0})A_{c}\geq 0\\ Then\;F_{n}\left(t_{1}\right)=0, \end{array}$$

where *σ*(*t*_0_) is the tensile strength of coal–rock materials.

In the simulation, the Mohr–Coulomb criterion is used to calculate and correct the tangential force of the interface contact element:

(10) $$\begin{array}{c} If\;F_{s}(t_{1})-F_{n}(t_{1})\times \tan\phi -c(t_{0})A_{c}\geq 0\\ Then\;F_{s}\left(t_{1}\right)=F_{n}\left(t_{1}\right)\times \tan\phi , \end{array}$$

where *ϕ* is the internal friction angle of coal–rock materials; $c(t_{0})$ is for the cohesion strength of coal–rock materials, respectively; Δu*_s_* is for the relative displacement in the tangential direction.

### 3.2. Numerical Modeling and Parameters

**Figure 4:**
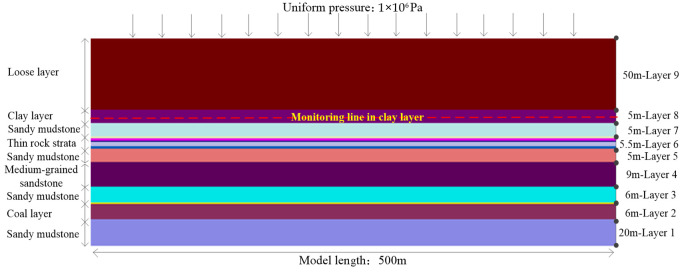
Numerical model of coal–rock layers.

According to the mining face parameters and geological conditions in this study, the dip angle of the coal seam in simulation is 0°. The bedrock thickness is 30 m, and the thickness of the aquiclude clay layer above the bedrock is 5 m. The thickness of aquifer alluvium above the clay layer is 50 m, and the uniform pressure of 10 × 10^6^ Pa is applied in the model top to simulate the geo-stress generated by about 400 m load layer. According to the geological conditions, numerical models of coal mining are established, as shown in Figure 4.

Referring to the measured mechanical parameters of bedrock layers in the 18,060 mining face according to the determination method of physical and mechanical properties of coal and rock [29], the input mechanical parameters of coal–rock strata in the simulation are shown in Table 3 [30].

### 3.3. Numerical Simulation Schemes

Coal mining is a complex three-dimensional evolution process, this study decomposes the complex process into two directions: the strike and inclined directions of the coal seam, respectively [31,32,33]. In this study, with the continuous mining of coal seam, the water-conducting fracture of the roof will evolve periodically along the advance direction of the mining face. Therefore, a 2D numerical simulation in the strike direction of the working face middle is carried out. Additionally, when the end structures of the mining face are destroyed, there will also be obvious separation and fracture structure above the roof strata, which is prone to cause a water/sand inrush disaster of the mining face. Therefore, a 2D simulation in the inclined direction of the mining face is carried out. 

For two mining technologies (layered mining and once full-height mining), the fracture, migration, and force characteristics of the coal–rock structure are compared, and the expansion laws of water-flowing fractured zone in the overlying strata are analyzed. Specifically, for the mining simulation of a coal seam strike, the mining length is 260 m, and the step-by-step excavation and calculation balance strategies are adopted. For the mining simulation in the inclined direction of a coal seam, the once-excavation and calculation balance strategies are adopted. For the once-mining full-height technology, the mining height of the coal seam is 6 m. In the layered mining simulation, the mining height of the top coal seam is 3.5 m, and the mining height of the bottom coal seam is 2.5 m. To study the influence of working face length in the inclined direction, the lengths of the top slicing working face are 180 m, 200 m and 220 m, respectively. The bottom working face is arranged inside 10 m of two ends of the top working face. The lengths are 160 m, 180 m and 200 m, respectively.

## 4. Simulation Result Analysis under Different Mining Technologies

The characteristic change of the mining working face is mainly concentrated in the initial pressure stage and periodic pressure stage. For the once-full-height mining, top slice mining, and bottom slice mining technologies, this section analyzes the simulation results of two-stage typical stope characteristic conditions.

### 4.1. Failure Characteristics of Overlying Strata in the Advancing Direction

**Figure 5:**
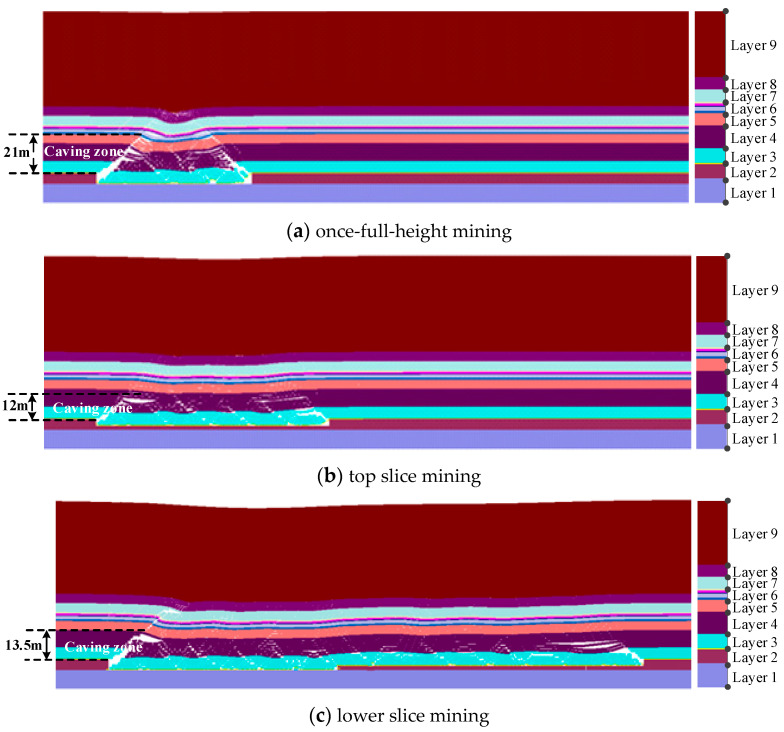
Coal–rock structure characteristics of advancing 80 m.

When the working face advances 80 m, the first roof pressure of the mining face occurs. In this stage, the rock structure characteristics under different mining technologies are shown in Figure 5.

From Figure 5a, under once mining full-height technology, the bedrock strata (layer 3–layer 7) above the coal seam generate the overall collapse and instability, and the collapse zone height is about 21 m. Above 30 m thick bedrock layers, the clay aquiclude (layer 8) produces obvious water-conducting fracture structures. From Figure 5b, during top-layered coal mining, the bedrock layers above the coal seam also produce an overall collapse and instability. The collapse zone develops to the medium-grained sandstone (layer 4), and the collapse zone height is about 12 m. The sandy mudstone (layer 7) beneath the clay aquiclude produces the separation phenomenon. However, above 30 m thick bedrock layers, clay aquiclude (layer 8) is in the deflection deformation area, and there is no obvious water-flowing fracture structure. From Figure 5c, during bottom layered mining, the loose rock structures in goaf are further compacted, and the caving zone develops to the top of medium sandstone (layer 4), and the caving zone height is about 13.5 m. Above the goaf and near the open-off cut position, the sandy mudstone (layer 7) below the clay aquiclude has an obvious separation phenomenon. However, the clay aquiclude (layer 8) is still in the deflection deformation area, and there is no obvious water-conducting fracture structure.

**Figure 6:**
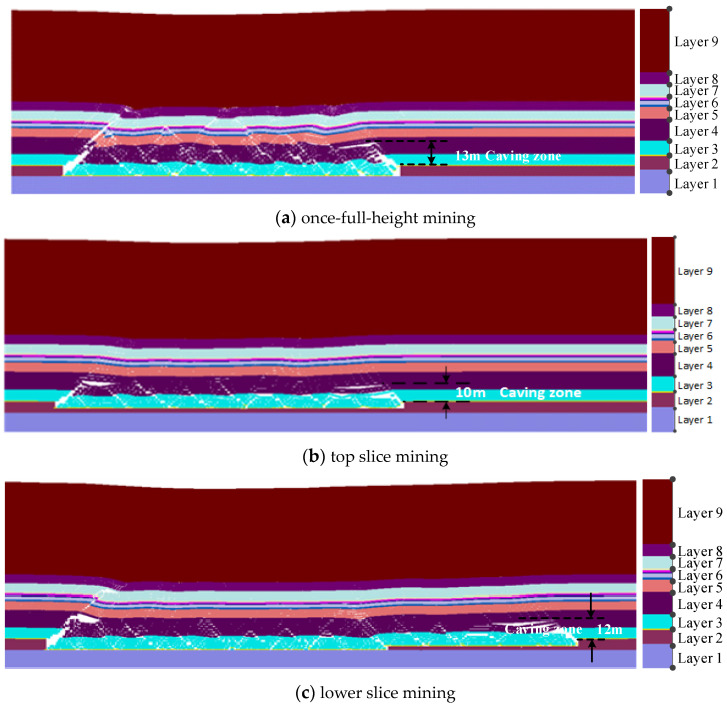
Coal–rock structure characteristics of advancing 220 m.

After the advancing distance of the coal mining face exceeds 220 m, the coal mining enters the normal mining stage of periodic pressure appearance. In this stage, the coal-rock structure characteristics are shown in Figure 6.

From Figure 6a, near the mining face, the rock layer with 13 m thickness is located in the caving zone under once full-height mining technology. Above the caving zone, the coal–rock layer presents the overlapping rock beam structure. Above the rock beam structure, rock layers are in the bending subsidence zone, and the clay aquiclude at 30 m above the coal seam has no water-conducting cracks. From Figure 6b, during the top layered mining, about 10 m thick strata above the coal seam are in the caving zone. Above the caving zone, the rock layers are in the deflection deformation zone, and there is no obvious crack in the clay layer. From Figure 6c, during the bottom layer mining, the goaf coal–rock structures near the working face are further compacted, the caving zone height develops to 12 m, and there is also no obvious fracture structure in clay aquiclude.

### 4.2. Failure Characteristics of Overlying Strata in the Inclination Direction

To explore and evaluate the failure and water-conducting characteristics of overlying strata in the mining face ends, the failure characteristics of overlying rock layers in the inclination direction of the mining face are analyzed under different mining technologies and working face lengths.

**Figure 7:**
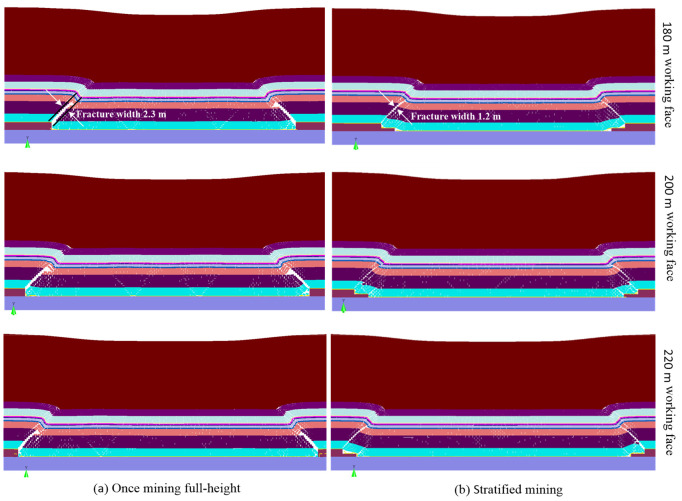
Coal–rock structure characteristics in the inclination direction.

From Section 4.1, after the advance distance of the mining face exceeds 220 m, the coal mining enters the periodic pressure appearance stage. In this stage, the overlying strata structure characteristics under different mining technologies are shown in Figure 7.

From Figure 7, the overlying rock structures in the middle of different length mining faces are in relatively compacted states; however, there are obvious separations at mining face ends due to the collapse of rock strata. Near two ends of the working face with different lengths, the lower rock layers present a cantilever rock beam structure, and the upper rock layers form a lap rock beam structure. Concretely, from Figure 7a, under once-full-height mining technology, the obvious separation of the caving zone occurs near the mining face ends, the separation height is about 2.3 m, and the rock layers above the caving zone are in the fracture development zone. Above 30 m thick bedrock, the clay aquiclude (layer 8) produces a certain degree of water-flowing fracture. After mining the coal seam (layer 2) of 180 m length working face, the clay layer produces a relatively weaker rupture and separation phenomenon; however, the clay aquiclude produces obvious fracture and separation after mining the coal seam of 200 m and 220 m length working faces. From Figure 7b, under stratified mining technology, the rock layers above the coal seam near working face ends are prone to present the lap rock beam structures. The obvious separation of the caving zone also occurs near mining face ends; however, the maximum separation height is only 1.2 m.

### 4.3. Mechanical Characteristics of Clay Aquiclude in the Advancing Direction

**Figure 8:**
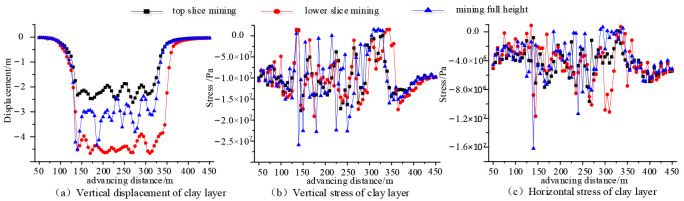
Mechanical characteristics of clay aquiclude in the advancing direction.

To further analyze the influences of mining disturbance on the water-resisting and sand-resisting abilities of the clay layer, the mechanical characteristics of clay aquiclude (layer 8) above the bedrock are explored and evaluated. The mechanical response characteristics of the clay layer above bedrock are shown in Figure 8.

From Figure 8a, after coal seam mining, the average settlement displacement of clay aquiclude (layer 8) under once-full-height mining technology is about 3 m, and the average settlement displacement of clay aquiclude (layer 8) is about 2.3 m under top-slicing mining technology. However, under bottom-slicing mining technology, the average settlement displacement of clay aquiclude is about 4.5 m. From Figure 8b and 8c, above the goaf, the vertical stress and horizontal stress at different positions of clay aquiclude show irregular fluctuation distributions. At different positions of stress curves of clay aquiclude, the stress fluctuation degree in once-full-height mining technology is greater than that of top-layer mining technology, and is slightly greater than that of bottom-layer mining technology. More specifically, from Figure 8b, after coal seam mining, the maximum value of vertical stress curves in clay aquiclude (layer 8) is about 26 MPa under once-full-height mining technology, the maximum value of vertical stress curves in clay aquiclude is about 20 MPa under top-layer mining technology, and the maximum value of vertical stress curves in clay aquiclude is about 18 MPa under bottom layer mining technology. From Figure 8c, the maximum horizontal stress at different positions of clay aquiclude (layer 8) is about 16.3 MPa under once-full-height mining technology, the maximum horizontal stress of clay aquiclude is about 12.1 MPa under top-layer mining technology, and the maximum horizontal stress of clay aquiclude is about 10.2 MPa under bottom layer mining technology.

### 4.4. Mechanical Characteristics of Clay Aquiclude in the Inclination Direction

**Figure 9:**
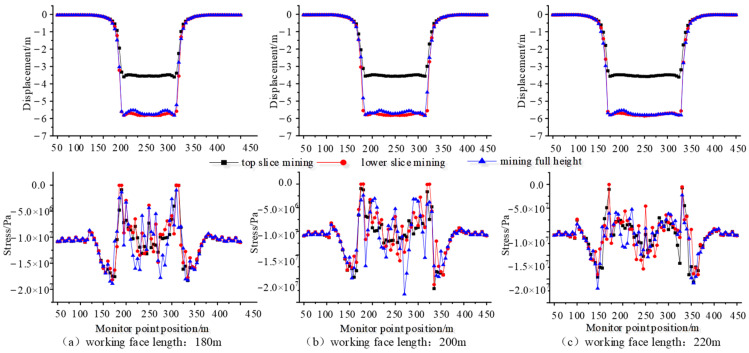
Mechanical characteristics of clay layer in the inclination direction.

For different working face lengths and mining technologies, in the inclination direction of the working face, the mechanical response characteristics of water-resisting and sand-resisting clay layer above 30 m thick bedrock are shown in Figure 9.

From Figure 9, under different mining technologies, the settlement displacement changes at different positions of clay aquiclude (layer 8) are as follows: the displacement of the clay layer near two ends of the mining face increases rapidly from the coal side to the goaf side, and remains stable in the middle area of goaf. The vertical displacement (3.5 m) of the clay layer during the top stratified mining is smaller than the vertical displacement (about 5.8 m) of the clay layer during the bottom stratified mining. Generally, the total vertical displacement of clay layer stratified mining technology is slightly larger than the vertical displacement (about 5.7 m) of once-mining full-height technology. However, there is no obvious influence on the settlement displacements of clay layers under different working face lengths. Further, the vertical stress variation at different positions of the clay layer is as follows: near both ends of mining face, the vertical stresses of clay layer are entirely larger than those of other positions under different working face lengths. With an increase in working face length, the vertical stresses of the clay layer near both ends of the mining face increase from 18 MPa to 20 MPa. For the middle of the 200 m mining face, the maximum vertical stresses (2.3 MPa) of the clay layer under once-mining full-height technology are greater than those (1.6 MPa) of bottom-layered coal mining, which are greater than those (1.0 MPa) of top-layered coal mining.

## 5. Discussion on Results of Different Research Methods

In this section, the empirical formulas, ground drilling, underground drilling and simulation detections are compared to analyze the caving zone heights of roof strata under different mining technologies. The obtained results are shown in Table 4. During once-full-height mining, the caving zone height of roof strata in simulation is in between the caving zone heights of two formulas in standard; during the layered mining, the caving zone heights of roof strata in the field detection are close to the caving zone heights of simulation detection. Those confirm the rationality of the research results.

From Section 4.1, in the initial pressure appearance stage of the mining face, the collapse zone height of overlying strata is about 21 m during once-full-height mining. For the layered mining technology, the collapse zone height is about 12 m during top-layer mining, the collapse zone height is about 13.5 m during bottom-layer mining. In the mining stage of periodic pressure appearance, the caving zone near the mining face side is only 13m during once-full-height mining, the caving zone height is about 10 m during the top layered mining, and the caving zone height develops to 12 m during the bottom layer mining. Therefore, the caving zone heights of overlying strata in the initial pressure appearance stage are larger than the mining stage of periodic pressure appearance. In the initial pressure appearance stage, the collapse zone height during once full-height mining is larger than the layered mining technology. However, in the mining stage of periodic pressure appearance, the collapse zone height during once full-height mining is slightly larger than the layered mining technology.

For the mechanical failure characteristics of clay aquiclude, the simulation detection results of advancing direction can represent the mechanical failure properties of the mining face middle. From Section 4.3, the average settlement displacement (3 m) of clay aquiclude during once full-height mining is smaller than the average settlement displacement (4.5 m) during the layered mining, which indicates the compacted degree of goaf rock structures during stratified mining is larger than that of once full-height mining. Moreover, the maximum vertical stress (26 MPa) of clay aquiclude during once full-height mining is larger than that (20 MPa) of layered mining, and the maximum horizontal stress (16.3 MPa) of clay aquiclude during once full-height mining is larger than that (12.1 MPa) of layered mining, which indicates the failure possibility of clay aquiclude during once-full-height mining is larger than that of stratified mining.

The simulation detection results of inclination direction can represent the mechanical failure properties of the mining face ends. From Section 4.2 and Section 4.4, the obvious separation of the caving zone occurs near two ends of the mining face, which is obviously larger than the mining face middle. The separation height during once-full-height mining (2.3 m) is larger than the separation height (1.2 m) during stratified mining. Moreover, the vertical displacement (5.8 m) of the clay layer during the stratified mining is slightly larger than the vertical displacement (5.7 m) during once-full-height mining. This indicates the mining face ends during once-full-height mining is more prone to produce the water inrush and sand inrush disaster of the mining face.

For the mechanical response of clay aquiclude above the thin bedrock and below the thick alluvium, due to the consolidation and compression deformation of alluvium itself, the thick alluvium forms a load on the bedrock layers during the mining process, thus aggravating the mining subsidence [34,35]. Under the large structural difference between bedrock and alluvium layers, the space disturbance of stratified mining is smaller than once-full-height mining, and the compacted degree of overlying rock is larger than that of once-mining full-height technology. Therefore, the settlement displacements of clay aquiclude during stratified mining are smaller than that of once-full-height mining technology. Because the mining disturbance of once-mining full-height technology is larger than that of layered mining technology, the maximum force of clay aquiclude during once-full-height mining is larger than that of layered mining technology, which more easily generates the fracture structure to inrush the water and sand of thick alluvium.

As the length of the mining working face is larger, the mining disturbance is larger, and the bedrock failure above the coal seam is more severe, so the vertical stresses of the clay layer near the mining face end increase from 18 MPa to 20 MPa with the increasing of the working face length. A large mining disturbance also easily generates more rock fracture, fissure, and separation of bedrock layer, which easily leads to the failure of clay aquiclude and form flowing-water channels. However, the influence of working face lengths on the settlement displacements of the clay layer is not obvious. Therefore, the influence of working face length on the water inrush and sand inrush disaster is larger than the influence of mining technology.

As a summary, the caving zone heights of roof strata under the layered mining technology are smaller than those of once-full-height mining, the coal–rock structures above the mining face are further compacted during stratified mining, so the rupture and separation phenomenon of bedrock layers are weaker than the once-full-height mining. Compared to once-full-height mining, the clay layer above 30 m thick bedrock is in the bending subsidence zone during stratified mining, there is no obvious water-flowing fractured zone near the two ends of the mining face.

As a result, the rupture and separation phenomenon of clay aquiclude above 30 m thick bedrock under the layered mining is weaker than those of once-full-height mining. Therefore, for coal seam mining in the thick loose layer and thin bedrock conditions, the layered mining technology can effectively reduce and prevent geological disasters such as water inrush and sand inrush of the mining face with different lengths.

## 6. Conclusions, Limitations, and Future Scope

To guarantee the safety mining of the coal seam in the thick loose layer and thin bedrock geological conditions, the separation and collapse of overlying strata under the mining disturbance are investigated by on-site observation, theoretical analysis, and simulation detection. The failure characteristics and mechanical response of the clay layer are used to evaluate the water-resisting and sand-resisting performance of the mining face. Some main conclusions can be drawn:

1. During once-full-height mining, the caving zone height of simulation detection is 21 m, which is in between the results (14.9 m and 24 m) of the two standards. The caving zone heights of on-site detection are 13.1 m and 14.65 m during the top-layer mining and bottom-layer mining. The caving zone heights of simulation detection are 12 m and 13.5 m during the top-layer mining and bottom-layer mining. Generally, the obtained caving zone heights of roof strata using three research methods are consistent to a certain degree, which confirms the rationality of the research results in this paper.
2. In the initial pressure appearance stage of the mining face, the maximum collapse zone height of overlying strata is about 21 m during once-full-height mining, and the collapse zone height is 13.5 m during layered mining. In the normal mining stage of periodic pressure appearance, the maximum caving zone near the mining face side is only 13 m during once-full-height mining, and the caving zone height develops to 12 m during the layered mining. Therefore, the caving zone heights of the initial mining stage are larger than the normal mining stage, and the collapse zone height during once-full-height mining is larger than the layered mining technology.
3. The maximum settlement displacement (5.8 m) of clay aquiclude during layered mining is slightly larger than that (5.7 m) of once full-height mining; however, the maximum stress (25 MPa) of clay layer during once full-height mining is greater than that (20 MPa) of layered mining. Therefore, the clay aquiclude during layered mining is prone to in the deflection deformation area, and there is no obvious water/sand inrush fracture structure. This indicates the failure possibility of clay aquiclude during once-full-height mining is larger than that of stratified mining.
4. For the influence of working face lengths, the separation and failure of overlying strata near mining face ends are larger than the mining face middle, the mining face ends are more prone to produce the water inrush and sand inrush disaster. The separation height during once-full-height mining (2.3 m) is larger than the separation height (1.2 m) during stratified mining. The vertical stresses of the clay layer near mining face ends increase from 18 MPa to 20 MPa with the increasing in working face length. However, the influence of working face lengths on the settlement displacements of the clay layer is not obvious.
5. Under once-full-height mining technology, the rock structures above the coal seam are prone to overall collapse and instability, and the clay layer above the bedrock easily produces obvious fracture structures. Under the stratified mining technology, the loose rock structure in the goaf is further compacted, the clay layer is in the deflection deformation, and there is no obvious fracture structure for water flowing and sand inrush. Therefore, for coal seam mining in thick loose layer and thin bedrock conditions, the stratified mining technology can effectively reduce and prevent geological disasters such as water inrush and sand inrush in the mining process of the working face.
6. In this study, to prevent the water/sand inrush disaster of mining working face, the influences of two mining technologies on the roof strata are investigated, and the caving zone heights of overlying strata are obtained by field detection, theoretical analysis, and simulation. Further, the deformation and failure of clay aquiclude are analyzed. Based on this, the possibility of water/sand inrush to the mining face is evaluated through the fracture structures of bedrock and clay layers, and an optimized mining technology is proposed to prevent the water/sand inrush disaster of the mining working face. This research can provide a guidance to ensure the mining safety. However, in the current study, the deformation and failure of clay aquiclude are only analyzed by simulation detection, the investigation results need to be certified by the engineering practice. Therefore, the investigation using advanced sensors to detect the mechanical characteristics of the clay layer still needs to be carried out. In the current paper, only the influences of two mining technologies on the clay layer failure are studied under specific geological conditions; therefore, the influences of more conditional changes also need to be investigated in the future.

## Figures and Tables

**Table 1:** The completion works of drilling and detection.

Final Hole Depth/m	Seen Bedrock Depth/m	Exposed Bedrock Thickness/m	Logging Depth/m	Drilling Hole Inclination/°
547.35	517.10	30.25	543.90	1

**Table 2:** Borehole observation results.

Borehole Number	1# Borehole	2# Borehole	3# Borehole	4# Borehole	Average Value
Caving zone height	15.26 m	14.32 m	14.68 m	14.32 m	14.65 m

**Table 3:** Coal and rock mechanics parameters.

Rock Strata	Thickness (m)	Density (Kg/m^3^)	Elastic Modulus (Pa)	Poisson’s Ratio	Tensile Strength (Pa)	Cohesion (Pa)	Internal Friction (°)
loose layer	50	1700	1.34 × 10^9^	0.42	1.30 × 10^5^	1.00 × 10^5^	30
clay layer	5	1700	1.34 × 10^9^	0.42	1.30 × 10^5^	1.00 × 10^5^	30
sandy mudstone	5	2697	8.37 × 10^9^	0.3	1.76 × 10^7^	2.14 × 10^6^	44
sandstone layer	1	2684	1.56 × 10^10^	0.3	2.65 × 10^7^	3.77 × 10^6^	44
mudstone layer	1	2740	9.11 × 10^9^	0.3	1.54 × 10^7^	1.59 × 10^6^	44
sandstone layer	0.5	2684	1.56 × 10^10^	0.3	2.65 × 10^7^	3.77 × 10^6^	44
sandy mudstone	2	2697	8.37 × 10^9^	0.3	1.76 × 10^7^	2.14 × 10^6^	44
sandstone layer	1	2684	1.56 × 10^10^	0.3	2.65 × 10^7^	3.77 × 10^6^	44
sandy mudstone	5	2697	8.37 × 10^9^	0.3	1.76 × 10^7^	2.14 × 10^6^	44
medium-grained sandstone layer	9	2684	1.56 × 10^10^	0.3	2.65 × 10^7^	3.77 × 10^6^	44
sandy mudstone	6	2697	8.37 × 10^9^	0.3	1.76 × 10^7^	2.14 × 10^6^	44
mudstone layer	0.5	2740	9.11 × 10^9^	0.3	1.54 × 10^7^	1.59 × 10^6^	44
coal seam	3.5	1426	2.80 × 10^9^	0.32	9.50 × 10^6^	2.60 × 10^6^	30
sandy mudstone	20	2697	8.37 × 10^9^	0.3	1.76 × 10^7^	2.14 × 10^6^	44

**Table 4:** Caving zone height comparison of different test methods and mining technologies.

Adopted Method	Mining Technology	Mining Height/m	Maximum Caving Zone Height/m
On-site detection	Top-layer mining	3.40	13.1
	Bottom-layer mining	5.80	14.65
Simulation detection	Top-layer mining	2.50	12
	Bottom-layer mining	3.50	13.5
Simulation detection	Once full-height mining	6.00	21
Formulas in standard	Once full-height mining	6.00	14.9~24.0

## Data Availability

Data are contained within the article.
